# Genomic and Functional Characterization of an *Alternaria brassicicola* Isolate Causing Black Spot Disease on Broccoli Leaves

**DOI:** 10.3390/life16071099

**Published:** 2026-06-30

**Authors:** Chunyan Qi, Rong Zeng, Guangqing Li, Liqing Zhang, Jian Yang, Peng Liu, Zhujie Xie

**Affiliations:** 1 State Key Laboratory for Quality and Safety of Agro-Products, Institute of Plant Virology, Ningbo University, Ningbo 315211, China; qcy20010130@163.com (C.Q.); nather2008@126.com (J.Y.); wood319@126.com (P.L.); 2 Eco-Environment Protection Research Institute, Shanghai Academy of Agricultural Sciences, Shanghai Key Laboratory of Protection Horticultural Technology, Shanghai 201403, China; zlq1985-345@163.com; 3 Horticultural Research Institute, Shanghai Academy of Agricultural Sciences, Shanghai Key Laboratory of Protection Horticultural Technology, Shanghai 201403, China; liguangqing0212@163.com

**Keywords:** *Alternaria brassicicola*, broccoli leaf spot, necrotrophic pathogen, effector, fungicide sensitivity, secretome, cell death suppression

## Abstract

*Alternaria brassicicola* is a necrotrophic fungal pathogen causing black spot disease on cruciferous crops worldwide. In this study, we comprehensively characterized the pathogenic isolate Ab0920a from diseased broccoli in Shanghai using morphological, phylogenetic, host range, fungicide sensitivity, genomic, and functional analyses. Pathogenicity tests on 27 cruciferous varieties revealed a broad host range with varying resistance levels. Fungicide sensitivity assays showed that fluxapyroxad (EC_50_ = 0.0695 µg/mL), prochloraz (0.0711 µg/mL), and difenoconazole (0.0863 µg/mL) were highly effective, whereas fluazinam was least effective. Genome-wide annotation identified 941 secreted proteins (8.95% of the proteome) and 237 candidate effectors, including 31 small cysteine-rich secreted proteins and 68 homologs of known virulence factors. Conserved effector-associated motifs (e.g., RXLR, [Y/F/W]xC) were detected, and carbohydrate-active enzyme CAZyme annotation revealed diverse families potentially involved in plant cell wall degradation. Functional validation using the pSUC2 yeast system confirmed that N-terminal signal peptides of tested effectors are competent for secretion. A PVX-based transient expression assay in Nicotiana benthamiana identified two effectors that suppress Bax-induced programmed cell death, suggesting their potential roles in modulating host immunity. Overall, this study provides comprehensive insights into the pathogen Ab0920a, offering resources for disease management and functional studies on necrotrophic fungal pathogenesis.

## 1. Introduction

*Alternaria brassicicola* is a prevalent necrotrophic fungal pathogen that causes black spot disease in economically valuable Brassicaceae crops such as broccoli, cabbage, cauliflower, mustard, and oilseed rape. Typical disease symptoms include dark leaf lesions, foliage blight, stem and petiole necrosis, and premature leaf senescence, ultimately leading to substantial yield loss. Disease incidence tends to rise sharply under conditions conducive to conidium formation and dispersal [1,2]. As the dominant pathogen infecting Brassicaceae plants, *A. brassicicola* frequently interacts with host tissues during pathogenic progression. Given the overlapping symptom phenotypes triggered by different Alternaria spp. in cruciferous crops, precise pathogen identification is critical for disease diagnosis, epidemiological surveillance, and the development of targeted management strategies [2,3].

Morphological observation of colonies and conidia remains the primary method for preliminary species discrimination. However, morphological characteristics are susceptible to variations induced by culture conditions and fungal developmental stages, resulting in limited identification accuracy. Accordingly, morphological identification is commonly combined with analysis of molecular markers, including the internal transcribed spacer, *Alternaria alternata* major allergen 1 (*Alt a1*), glyceraldehyde-3-phosphate dehydrogenase (*GPD*), Adenosine triphosphatase (*ATPase*) and calmodulin (*CAL*) genes, to improve the resolution of interspecific classification within *Alternaria* spp. [2,3,4,5]. Pathogenicity assays further verify the pathogenicity of the isolated strains by fulfilling Koch’s postulates and associating fungal isolates with observed disease symptoms, which are indispensable for diagnosing leaf spots and necrotic disorders in field-cultivated cruciferous vegetables.

Infection of Brassicaceae hosts by *A. brassicicola* is a highly complex, multi-phase process that involves dynamic molecular crosstalk between the fungus and the plant. Upon landing on the leaf surface, spores adhere, germinate, and differentiate into melanized appressoria, which generate mechanical pressure and secrete cutinases to breach the cuticle and epidermal cell walls [6]. Following penetration, the fungus rapidly colonizes the mesophyll, deploying an arsenal of cell wall-degrading enzymes (e.g., pectinases, cellulases, and xylanases) and phytotoxins, such as brassicicolin A, depudecin, and other host-selective toxins, that induce massive necrosis and facilitate nutrient leakage from damaged cells [7,8]. In addition, *A. brassicicola* secretes putative effector proteins that suppress host basal immunity and modulate plant metabolism, as revealed by transcriptomic profiling of in planta-expressed genes [6]. Concurrently, Brassicaceae plants activate a multilayered defense network: preformed barriers such as epicuticular waxes and cell wall lignification [9], followed by induced responses including phytoalexin accumulation (e.g., camalexin), reactive oxygen species bursts, and callose deposition [10]. Hormonal signaling mediated by jasmonic acid, salicylic acid, and ethylene orchestrates transcriptional reprogramming of thousands of defense-related genes, often with antagonistic or synergistic interactions that fine-tune the response [11,12]. The outcome of infection is determined by the timing, amplitude, and spatial coordination of these fungal virulence factors and host defense components, underscoring the necessity of integrating genomic, transcriptomic, and functional assays to dissect the molecular dialogue and identify durable disease management strategies.

Necrotrophic fungi, such as *A. brassicicola*, destroy host tissues to obtain nutrients, a process that relies heavily on the secretion of a diverse array of proteins. Secreted proteins, particularly those that function at the plant–pathogen interface, are key determinants of necrotrophic pathogenesis. Among these, the candidate effector molecules are of particular interest. Unlike the well-understood effectors of biotrophs, which suppress host immunity, necrotrophic effectors often function to induce host cell death, degrade cellular components, or neutralize plant-derived antimicrobial compounds [13,14]. In *A. brassicicola*, the capacity for efficient secretion is critically linked to successful infection. Previous studies have indicated that in planta secretion of fungal proteins is integral to its nutrient acquisition strategy, and that unfolded protein response signaling, which regulates secretory pathway capacity, is associated with efficient leaf and silique colonization and full virulence. These observations provide a strong biological rationale for prioritizing secretome and candidate effectors during genome analysis.

Candidate fungal effectors are commonly predicted from genomic data using combined criteria, including the presence of an N-terminal signal peptide, absence of additional transmembrane domains, relatively small protein size, cysteine enrichment, and lack of obvious housekeeping metabolic domains [15,16]. Computational screening, however, does not prove the effector function, but provides a tractable set of candidates for downstream experimental validation. Comparative annotation against pathogen–host interaction databases can further identify homologs of experimentally characterized virulence-associated genes, thereby placing newly analyzed isolates within a broader pathogenicity framework [17]. Candidate effectors are thus considered a specialized subset of the broader secretome, with a primary role in modulating plant–pathogen interactions.

Genome-wide functional annotation facilitates the identification of conserved protein domains, functional motifs, carbohydrate-active enzymes (CAZyme), and genes homologous to verified pathogen–host interaction (PHI) factors [17,18,19], all of which are directly relevant to virulence. For example, conserved LysM (lysin motif) domains in fungal effectors bind host chitin to suppress chitin-triggered immunity—a function demonstrated in the necrotrophic fungus *Botrytis cinerea* [20]. LysM effectors are widely distributed in fungal plant pathogens and their ability to interfere with host immune responses has been shown to contribute to virulence in various species. CAZymes, including cellulases, pectinases, and hemicellulases, degrade plant cell wall polymers to enable tissue penetration and nutrient acquisition—a hallmark of necrotrophic pathogens like *A. brassicicola*. The role of CAZymes in fungal pathogenesis has been extensively reviewed and their contribution to virulence is well documented across diverse fungal pathogens [21]. Additionally, homology searches against curated pathogen–host interaction databases such as PHI-base can identify candidate genes sharing similarity with known virulence factors, offering immediate clues to pathogenic mechanisms [17]. Together, these genome-wide annotations provide a powerful foundation for prioritizing functional categories most likely involved in host colonization, stress adaptation, and virulence, thereby guiding downstream experimental validation.

Experimental validation is indispensable to confirm the role of the predicted effectors. Heterologous secretion assays, such as yeast signal peptide trap systems, are frequently used to assess whether predicted signal peptides can mediate protein secretion outside the native fungal context. Furthermore, in planta transient expression assays, including expression in *Nicotiana benthamiana* leaves or virus-based expression systems, can be used to evaluate whether candidate proteins induce necrosis, suppress programmed cell death, or otherwise alter host immune responses [6,22,23,24]. These assays provide functional evidence linking genome-based predictions to biological activity during host interactions.

Chemical control remains an important component of Alternaria disease management in vegetable and oilseed production. However, fungicidal efficacy may vary among active ingredients and pathogen populations, and repeated use of fungicides with the same mode of action can select for resistant isolates [8,25]. Therefore, laboratory fungicide sensitivity assays are useful to estimate the relative inhibitory activities of different compounds before field deployment. This approach is consistent with the broader role of chemical control in crop disease management, and the practical importance of fungicide sensitivity assays for evaluating pathogen responses to fungicides. Integrating fungicide sensitivity data with accurate pathogen identification can reduce the risk of control failure and support sustainable disease management [9,26,27,28].

In the present study, an Alternaria isolate associated with broccoli leaf spotting and petiole necrosis in Shanghai was investigated using an integrated pathological, molecular, and genomic approach. The objectives were to: (1) identify the causal pathogen through morphological observation, molecular marker analysis, and pathogenicity testing; (2) evaluate its sensitivity to selected fungicides; (3) annotate genome-wide functional features, including conserved domains, and pathogen–host interaction homologs; and (4) predict and provide preliminary validation of candidate secreted effectors, with a focus on understanding their potential role in necrotrophic pathogenesis. Together, these analyses provide information for disease control and genomic resources to understand the potential virulence mechanisms of *A. brassicicola* in broccoli.

## 2. Materials and Methods

### 2.1. Isolation, Identification and Pathogenicity Assay of Pathogen Isolate Ab0920a from Diseased Broccoli

Diseased broccoli leaf samples were collected from the broccoli breeding baseon 20 September 2023. Pathogens were isolated according to the method described by Kaur [8]. Twenty fungal isolates with similar morphological characteristics were obtained, and isolate Ab0920a was selected as the representative strain for subsequent experiments. Single-spore isolation was used to obtain pure cultures with uniform genetic backgrounds. The purified isolate Ab0920a was cultured on potato dextrose agar (PDA) at 25 °C. Colony morphology, color, texture, and growth rate were recorded. Microscopic characteristics, including conidia and reproductive structures, were observed for preliminary identification of the pathogen. The pathogenicity of the isolate, Ab0920a, was confirmed by spore spray inoculation. Host plants were *B. oleracea* var. *italica* cv. ‘Hu-Lv 001’ at the 4–6 leaf stage. A 1 × 10^6^ spores/mL suspension of Ab0920a was uniformly sprayed onto healthy leaves until runoff; sterile water served as the control. Each treatment had three biological replicates. Inoculated plants were incubated in a humid chamber (25 °C, 90% relative humidity) for 24 h in the dark, then transferred to normal growth conditions (25 °C, 16 h light/8 h dark, 70% RH). Disease symptoms were recorded daily for seven days post-inoculation (dpi). To satisfy Koch’s postulates, fungi were reisolated from symptomatic tissues and their morphological characteristics were confirmed to be consistent with the original isolate Ab0920a.

### 2.2. Multilocus Gene Sequencing and Phylogenetic Analysis

Genomic DNA was extracted from fresh mycelia using a modified CTAB method. Four gene regions (*Alt a1*, *ATPase*, *CAL*, *GPD*) were amplified (Table S1) in a 25 μL PCR reaction (DNA template, primers, 2× PCR Mix). Thermal cycling conditions were as follows: initial denaturation at 94 °C for 5 min; 35 cycles of 94 °C for 20 s, 56 °C for 20 s, 72 °C for 30 s; and final extension at 72 °C for 5 min. PCR products were purified and cloned into the Vazyme ClonExpress II vector (Vazyme Biotech, Nanjing, China), and positive clones were sequenced bidirectionally using the M13F/M13R primers (five clones per gene for accuracy). The confirmed sequences were submitted to GenBank. Consensus sequences were aligned with MUSCLE (v5) [trimAl (v1.5.1) for poorly aligned regions] [29,30]. Multilocus phylogenetic analysis was conducted using IQ-TREE (v3) and the maximum likelihood method with 1000 bootstrap replicates [31]. Reference sequences of *Alternaria* spp. and the outgroup *Stemphylium vesicarium* were included, and all sequences used are listed in Table 1. The phylogenetic tree was visualized using FastTree (v2.2) [32].

### 2.3. Pathogenicity Assay of the Pathogen on Brassicaceae Crops

Thirty cruciferous varieties (5 broccoli, 6 pak choi, 4 Chinese cabbage, 3 cauliflower, 3 Chinese broccoli, 3 white radish, 2 shepherd’s purse, 1 cabbage; Table 2) were used. Seeds were surface sterilized (75% ethanol for 30 s, then rinsed with sterile water) and sown in sterilized peat moss, vermiculite, and perlite (3:1:1, *v*/*v*/*v*). Seedlings were grown at 26 ± 1 °C (12 h light/dark, 300 μmol·m^−2^·s^−1^, 60 ± 5% RH). At the 3–6 leaf stage, seedlings were spray-inoculated with 5 mL of Ab0920a spore suspension (1 × 10^6^ conidia·mL^−1^ in 0.05% Tween 80); controls received 0.05% Tween 80. Each variety had three biological replicates (10 seedlings/replicate), with a 24 h post-inoculation incubation at 90 ± 5% RH before the plants were returned to original conditions. Disease severity was assessed on a 0–9 scale where 0 = no visible lesions; 1 = lesions limited to leaf area, <5% of leaf area; 3 = 5–10% of leaf area affected; 5 = 11–25%; 7 = 26–50%; and 9 = >50% with severe chlorosis/necrosis. Disease index (DI) was calculated as: DI (disease index) = [∑(Grade × Number of plants in grade)/(Total plants × Highest grade)] × 100. Host responses were categorized as follows: highly resistant (HR, DI = 0), resistant (R, 0 < DI ≤ 10), moderately resistant (MR, 10 < DI ≤ 30), moderately susceptible (MS, 30 < DI ≤ 50), susceptible (S, 50 < DI ≤ 70), and highly susceptible (HS, DI > 70).

### 2.4. Sensitivity to Fungicides

The mycelial growth inhibition method was adopted as described by Kaur [11] to determine the in vitro efficacy of nine fungicide active ingredients against the isolate Ab0920a. The tested strain was cultured on PDA plates in darkness at 26 °C for 7 d, and 6 mm diameter mycelial plugs were punched from the colony edges and inoculated in the center of fungicide-amended PDA plates with the mycelium side facing downward. All fungicides were dissolved in dimethyl sulfoxide (DMSO) to prepare stock solutions with the corresponding concentrations and then added to the PDA. The concentration gradients were set based on preliminary experiments. The concentration gradients of fluopyram, fluxapyroxad and prochloraz were 0.08, 0.16, 0.32, 0.64, 1.28 and 2.56 μg/mL; those of hexaconazole, epoxiconazole, tebuconazole and iprodione were 0.10, 0.20, 0.40, 0.80 and 1.60 μg/mL; those of difenoconazole were 0.032, 0.064, 0.128, 0.256, 0.512 and 1.024 μg/mL; and those of fluazinam were 0.10, 0.30, 0.90, 2.70, 8.10 and 24.30 μg/mL. PDA plates supplemented with 100 μL DMSO served as the control group. Each treatment was replicated six times. After incubation in darkness at 26 °C for 7 d, colony diameters were measured using the cross method, and the mycelial growth inhibition rate was calculated using the following formula: Mycelial growth inhibition rate = (Colony diameter of control group − Colony diameter of treatment group)/(Colony diameter of control group − 6 mm) × 100%.

The experimental data were processed using Office software (Excel 365), and linear regression analysis was performed using DPS (v16.05) software [33]. The regression equation slope, standard error (SE), chi-square value (χ^2^), degree of freedom, median effective concentration (EC_50_) and 95% confidence limits were calculated.

### 2.5. Prediction and Functional Characterization of Candidate Effectors in A. brassicicola

The annotated genome of *A. brassicicola*, comprising 10,514 protein-coding genes, was retrieved from the Joint Genome Institute (JGI) database [34]. All the protein sequences were used for subsequent secretome and effector predictions. All sequence-processing and filtering steps were performed using custom Python scripts (Figure S1). Candidate effectors were predicted via a multi-step filtering pipeline: SignalP 6.0 with the “eukaryotes” model (D-cutoff = 0.5) was used to detect canonical N-terminal signal peptides [35]. Proteins with ≥1 predicted transmembrane helix were excluded using TMHMM 2.0 [36]. TargetP 2.0 with the “plant” model (reliability class RC ≤ 3) was employed to filter out proteins containing mitochondrial targeting signals [37]. Secreted proteins passing through the filters were subjected to effector prediction using EffectorP 3.0, a specialized tool for fungal effector identification. Proteins classified as “effector” with a confidence score ≥ 0.7 were designated as candidate effectors [15].

Pfam domain analysis, InterProScan 108.0, with the Pfam database (v38.1) was used to identify pathogenesis-related domains, including common in fungal extracellular membrane (CFEM), Lysin Motif (LysM), necrosis-inducing protein (NlP), Phox homology (PX), Crinkler (CRN), Chitin_bind1, FAD-binding (FAD), and common central domain of tyrosinase (CCDT) [38,39]. Small cysteine-rich secreted proteins (SCRPs) were selected from candidate effectors based on molecular weight ≤ 20 kDa and cysteine residue number ≥ 4. The candidate effectors were aligned to the pathogen–host interactions database (PHI v4.12) using BLASTp (E-value ≤ 1 × 10^−5^). Hits with ≥30% sequence identity and ≥50% query coverage were considered significantly homologous to known virulence factors [17]. Additionally, known fungal effector motifs (RXLR, [Y/F/W]xC, G[I/F/Y][A/L/S/T]R, [L/I]xAR, YxSL[R/K], and LFLAK) were screened using regular expressions in BioEdit (v7.2.5). CAZymes were annotated using the dbCAN3 meta server using the “fungi” model [40]. Hits supported by at least two of the three methods (HMMER, dbCAN-sub, and DIAMOND) were retained and classified according to the CAZyme database into the families auxiliary activities (AA), carbohydrate-binding modules (CBM), carbohydrate esterases (CE), glycoside hydrolases (GH), glycosyltransferase (GT), and polysaccharide lyases (PL) [19].

### 2.6. Secretion Validation via the pSUC2 Yeast System

The secretions of seven candidate effectors was validated using the pSUC2 system (with SUC2 invertase as a reporter) [41]. The signal peptide-encoding regions of the candidate effectors were PCR-amplified and cloned into the pSUC2 vector (native signal peptide deleted); pSUC2-Mg87 and pSUC2-Avr1b (known secreted protein signal peptide [42]) served as the negative and positive controls, respectively. The DNA sequences of the seven candidate effectors and the primers used for amplification are provided in Table S2 and Table S3, respectively. The three vectors were transformed into the sucrose utilization-defective yeast strain YTK12 using LiAc/ssDNA/PEG, and transformants were selected on SD-Leu medium [43,44]. Selected clones were inoculated onto YPRAA medium (1% yeast extract, 2% peptone, 2% raffinose, 2 µg/mL antimycin A; Coolaber, Beijing, China) containing sucrose as the sole carbon source; growth indicated invertase secretion. 2,3,5-Triphenyltetrazolium chloride (TTC) staining confirmed activity (red colonies = secretion-positive). Additional media (CMD-W and YPRA) and sucrose were used for yeast culture. Candidate effectors were identified as secretory proteins only if the transformants grew on YPRAA and showed positive TTC staining, with consistent control performance.

### 2.7. Pvx-Based Functional Validation of Effector Regulation on Bax-Induced Cell Death

Seven candidate effector genes were amplified from pathogen cDNA using gene-specific primers flanked by *EcoR*I and *Xho*I restriction sites. The PCR products were purified, double-digested with *Eco*RI and *Xho*I, and ligated into the pGR107 PVX binary vector [35]. Recombinant plasmids were transformed into Escherichia coli DH5α and verified by restriction enzyme digestion and Sanger sequencing. The confirmed pGR107-effector constructs, the GFP-expressing vector (pGR107-GFP, negative control), and the Bax-expressing vector (pGR107-Bax, positive control) were separately introduced into *Agrobacterium tumefaciens* GV3101 by the freeze–thaw method. Positive transformants were confirmed by colony PCR. Four-week-old *N. benthamiana* plants were grown under a 16 h light/8 h dark photoperiod at 25 °C and 70% relative humidity. Agrobacterium cultures carrying the respective vectors were harvested, resuspended in infiltration buffer (10 mM MgCl_2_, 10 mM MES, pH 5.6, 200 μM acetosyringone) to an OD_600_ of 0.5, and incubated in the dark for 3 h. For single-effector assays, the bacterial suspension was infiltrated into the abaxial side of *N. benthamiana* leaves. For co-expression assays, the effector-containing culture and the Bax-containing culture were mixed at a 1:1 (*v*/*v*) ratio prior to infiltration. The following controls were included: empty pGR107 alone (negative control), pGR107-GFP (negative control for cell death), pGR107-Bax (positive control for strong cell death), co-infiltration of GFP and Bax (to confirm that an irrelevant protein does not interfere with Bax-induced death), and empty pGR107 co-infiltrated with Bax (as an additional positive control). Three biological replicates were performed for each treatment. Infiltrated leaves were photographed at 5–7 dpi.

## 3. Results

### 3.1. Disease Symptoms in Broccoli Leaves in the Field

**Figure 1:**
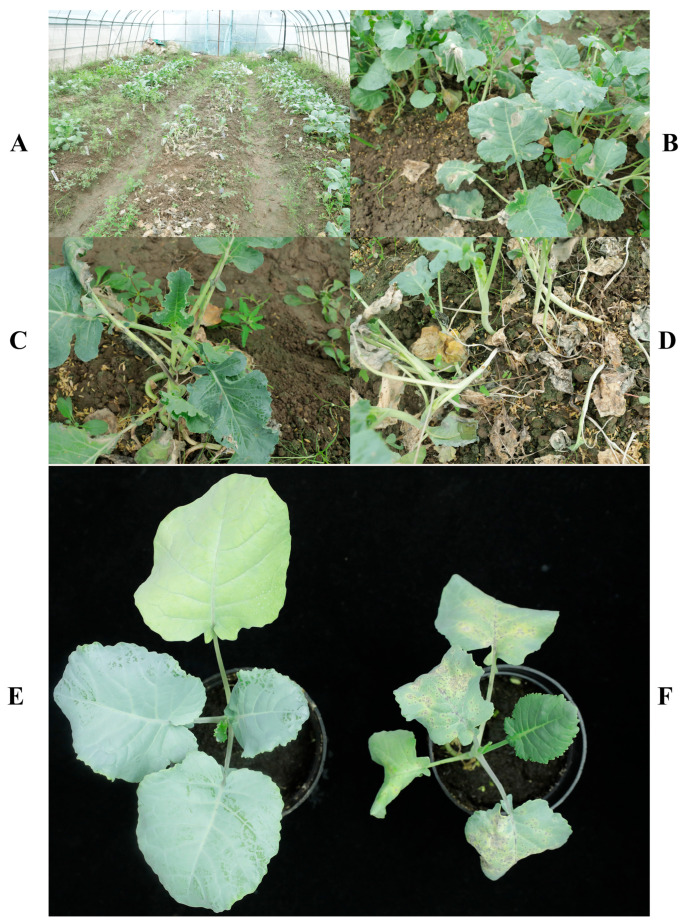
Symptoms of black spot disease in the field and pathogenicity assay of Ab0920a on broccoli plants. (**A**) Symptoms of black spot disease occurring in the field; (**B**) symptoms of black spot disease on broccoli leaves; (**C**) symptoms of black spot disease on broccoli petioles or stems; (**D**) symptoms of leaf withering and abscission caused by black spot disease; (**E**) control group: broccoli plant sprayed with sterile distilled water (no disease symptoms observed); (**F**) inoculated group: broccoli plant at 5 days post-inoculation (dpi) with 1 × 10^6^ conidia/mL spore suspension of Ab0920a (spray inoculation method), showing typical black spot disease symptoms (dark brown to black lesions on leaves and visible chlorosis around infected areas).

Typical disease symptoms were observed in most of the germplasm resources in the seedling field, with a field disease incidence of approximately 85%. At the initial stage of infection, circular to subcircular lesions appeared on the leaves, whereas elongated, fusiform, dark lesions formed on the stems. As the disease progressed, the lesions expanded and coalesced. In later stages, entire leaves became necrotic and were shed, and abundant dark spore masses developed around the central lesion areas (Figure 1A–D). Spray inoculation with isolate Ab0920a (1 × 10^6^ spores/mL) on 4–6 leaf-stage broccoli plants caused typical necrotic leaf spots at 3 days post-inoculation dpi. Lesions expanded and coalesced by 5 dpi, leading to severe blighting and wilting at 7 dpi. No symptoms were observed on water-sprayed controls (Figure 1E,F). The fungus was reisolated from symptomatic tissues, and its morphology matched the original isolate Ab0920a, fulfilling Koch’s postulates.

### 3.2. Characteristics of Ab0920a

**Figure 2:**
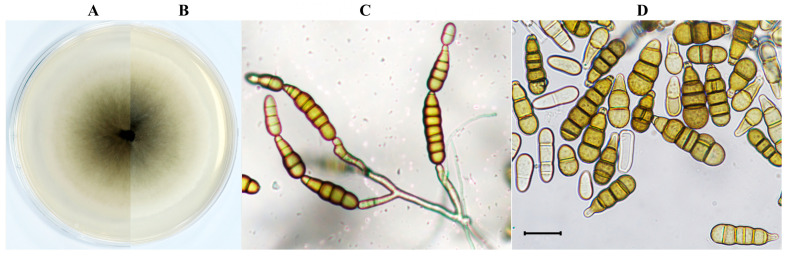
Colony morphology of Ab0920a cultured on PDA medium in the dark for 7 days and morphological characteristics of conidia. (**A**) Colony reverse; (**B**) colony obverse; (**C**) conidia formed in chains on the conidiophores; (**D**) mature conidia with varying numbers of transverse septa. Scale bar, 20 μm.

The purified isolate Ab0920a was cultured on PDA at 25 °C. The colonies initially developed abundant white cottony aerial mycelia. After 3–5 d of incubation, the colony color gradually deepened from white to yellowish-brown or dark brown, and conidia were produced in the aerial hyphae. The colony surface became slightly velvety to powdery as sporulation progressed (Figure 2A,B). Microscopic observations showed that isolate Ab0920a produced brown to yellowish-brown conidia, which were obclavate to ellipsoid in shape, with smooth to slightly verrucose walls. Mature conidia ranged 8.6–55.8 μm in length and 8.3–39.3 μm in width. The conidia were muriform, with 2–8 transverse septa, 0–4 longitudinal septa, and occasionally 1–3 oblique septa. A short conical beak was commonly present at the apex of mature conidia, whereas immature conidia were lighter in color, shorter, and had fewer septa (Figure 2C,D). These morphological characteristics were consistent with those of species in the genus *Alternaria*.

### 3.3. Multilocus Phylogeny and Molecular Identification of Isolate Ab0920a

For the molecular identification of isolate Ab0920a, four loci (*Alt a1*, *ATPase*, *CAL*, and *GPD*) were amplified and sequenced. To ensure sequence accuracy, five independent clones of each gene were bidirectionally sequenced and identical sequences were obtained for all clones of the same gene. The resulting consensus sequences were deposited in GenBank under accession numbers PZ399336 (*Alt a1*), PZ399337 (*ATPase*), PZ399338 (*CAL*), and PZ399339 (*GPD*).

**Figure 3:**
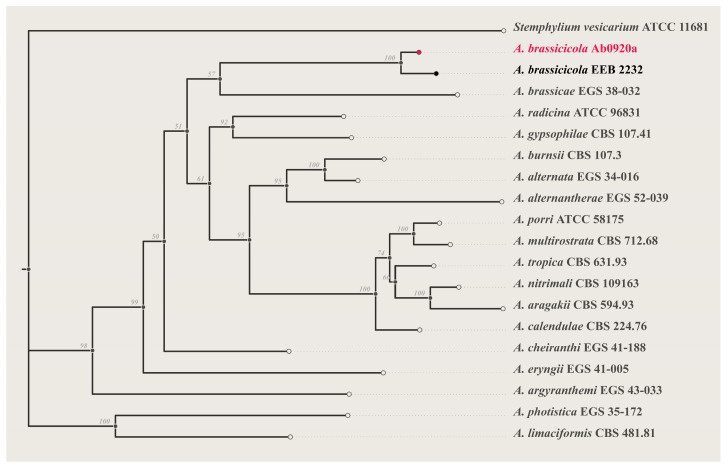
Phylogenetic tree based on multilocus sequence analysis showing the relationship between Ab0920a and related *Alternaria* species.

Multilocus phylogenetic analysis was performed using concatenated sequences of the four genes. The maximum likelihood tree, constructed with 1000 bootstrap replicates and rooted using *S. vesicarium* as the outgroup, revealed that isolate Ab0920a formed a distinct, well-supported clade (bootstrap value = 100) together with the reference strain *A. brassicicola* EEB 2232. Based on the molecular data, the isolate was identified as *A. brassicicola* (Figure 3).

The tree was constructed using the maximum likelihood method. Bootstrap values (≥50%) are shown at the nodes. *S. vesicarium* ATCC 11,681 was used as the outgroup.

**Table 1:** Species used for phylogenetic analyses in this study, their sources, and their GenBank accession numbers.

Species	Source	*GPD*	*Alt a1*	*ATPase*	*CAL*
*A. alternantherae*	EGS 52-039	JN383477 ^a^	JN383511 ^a^	JQ671892 ^b^	JQ646226 ^b^
*A. alternata*	EGS 34-016	AY278808 ^c^	AY563301 ^d^	JQ671874 ^b^	JQ646208 ^b^
*A. argyranthemi*	EGS 43-033	AY562400 ^d^	AY563280 ^d^	JQ671784 ^b^	JQ646118 ^b^
*A. burnsii*	CBS 107.3	JQ646305 ^b^	KP123967 ^b^	JQ671860 ^b^	JQ646194 ^b^
*A. brassicae*	EGS 38-032	AY562414 ^d^	AY563309 ^d^	JQ671847 ^b^	JQ646181 ^b^
*A. brassicicola*	EEB 2232	AY278813 ^c^	AY563311 ^d^	JQ671843 ^b^	JQ646177 ^b^
*A. cheiranthi*	EGS 41-188	AY278802 ^c^	AY563290 ^d^	JQ671830 ^b^	JQ646164 ^b^
*A. eryngii*	EGS 41-005	AY562416 ^c^	AY563313 ^d^	JQ671844 ^b^	JQ646178 ^b^
*A. gypsophilae*	CBS 107.41	JQ646304 ^b^	JQ646387 ^b^	JQ671859 ^b^	JQ646193 ^b^
*A. limaciformis*	CBS 481.81	JQ646277 ^b^	JQ646368 ^b^	JQ671798 ^b^	JQ646132 ^b^
*A. photistica*	EGS 35-172	AY562402 ^d^	AY563282 ^d^	JQ671807 ^b^	JQ646141 ^b^
*A. porri*	ATCC 58175	AY278806 ^c^	AY563296 ^d^	JQ671901 ^b^	JQ646235 ^b^
*A. radicina*	ATCC 96831	AY278797 ^c^	AY563286 ^d^	JQ671851 ^b^	JQ646185 ^b^
*A. tropica*	CBS 631.93	JQ646354 ^b^	JQ646438 ^b^	JQ671930 ^b^	JQ646264 ^b^
*A. nitrimali*	CBS 109163	JQ646358 ^b^	JQ646442 ^b^	JQ671934 ^b^	JQ646268 ^b^
*A. multirostrata*	CBS 712.68	JQ646362 ^b^	JQ646446 ^b^	JQ671938 ^b^	JQ646272 ^b^
*A. calendulae*	CBS 224.76	JQ646330 ^b^	JQ646414 ^b^	JQ671895 ^b^	JQ646229 ^b^
*A. aragakii*	CBS 594.93	JQ646359 ^b^	JQ646443 ^b^	JQ671935 ^b^	JQ646269 ^b^
*Stemphylium vesicarium*	ATCC 11681	AY278823 ^c^	AY563277 ^d^	JQ671770 ^b^	JQ646104 ^b^
*A. brassicicola*	Ab0920a	PZ399339	PZ399336	PZ399337	PZ399338

Sequences that were generated in this study appear in boldface. ^a^ Lawrence et al. 2012 [4]; ^b^ Lawrence et al. 2013 [2]; ^c^ Pryor and Bigelow 2003 [45]; ^d^ Hong et al. 2005 [5].

### 3.4. Pathogenicity of A. brassicicola Ab0920a on Different Brassicaceae Crops

The pathogenicity of isolate Ab0920a was evaluated in 27 cruciferous varieties, and distinct disease responses were observed across the tested hosts at 7 dpi. No symptoms developed in control plants treated with 0.05% Tween 80, confirming that the lesions observed in the inoculated plants were caused by the isolate.

Based on the calculated DI values, the 27 varieties were categorized into six reaction types: highly resistant (HR, DI = 0), resistant (R, 0 < DI ≤ 10), moderately resistant (MR, 10 < DI ≤ 30), moderately susceptible (MS, 30 < DI ≤ 50), susceptible (S, 50 < DI ≤ 70), and highly susceptible (HS, DI > 70). Among them, no variety was classified as HR; 12 varieties were R, such as Lvxiu 100, Qingyang 90, and Qingyang 60; 10 varieties were MR, including Aijiao Suzhouqing, Aijiao Qingcai, Lixiang Roucai et al.; three varieties were MS, such as Xinxuan Jingfeng No. 1, Qingfeng 120, and 8132; two varieties were S, including Chunfeng and Qinghuacai 75; and no variety was HS. Notably, host responses varied within and among crop species. For example, Chinese cabbage varieties exhibited reactions ranging from HR to HS, whereas cauliflower and cabbage varieties were predominantly MR to MS. White radish, shepherd’s purse, and pak choi varieties generally displayed moderate susceptibility. These findings demonstrate that isolate Ab0920a has a broad host range among cruciferous crops, with significant variations in resistance levels among different varieties (Table 2).

**Table 2:** Disease index (DI) and resistance ratings of 27 cultivars.

Cultivar	Category	DI	Resistance
Chunfeng	*B. oleracea* var. *italica*	65.87	susceptible
Xinxuan Jingfeng No. 1	*B. oleracea* var. *italica*	33.33	moderately susceptible
Qinghuacai 75	*B. oleracea* var. *italica*	57.78	susceptible
Lvxiu 100	*B. oleracea* var. *italica*	0.69	resistant
Qingfeng 120	*B. oleracea* var. *italica*	34.26	moderately susceptible
Qingyang 90	*B. oleracea* var. *botrytis*	3.70	resistant
Qingyang 60	*B. oleracea* var. *botrytis*	1.67	resistant
Aijiao Suzhouqing	*B. rapa* subsp. *chinensis*	27.41	moderately resistant
Aijiao Qingcai	*B. rapa* subsp. *chinensis*	11.11	moderately resistant
Lixiang Roucai	*B. rapa* subsp. *chinensis*	26.80	moderately resistant
Suxiaofei	*B. rapa* subsp. *chinensis*	8.47	resistant
Changgan Baicai	*B. rapa* subsp. *chinensis*	27.78	moderately resistant
Lvxiu Siji Jianye Youqing Tiancaixin	*B. rapa* subsp. *chinensis*	9.88	resistant
Yangzhou Yuanbai Luobo	*Raphanus sativus*	25.00	moderately resistant
Yibanhong Luobo	*R. sativus*	6.48	resistant
Xinglü No. 1 Ganlan	*B. oleracea* var. *capitata*	17.17	moderately resistant
8132	*B. oleracea* var. *alboglabra*	37.50	moderately susceptible
Qiangli 50	*B. oleracea* var. *alboglabra*	9.52	resistant
Lüxian Jielan	*B. oleracea* var. *alboglabra*	2.08	resistant
Chunfeng Xiulü	*B. oleracea* var. *alboglabra*	10.42	moderately resistant
Huangya 14	*B. oleracea* var. *alboglabra*	6.35	resistant
Siji Kuai Cai	*B. oleracea* var. *alboglabra*	22.22	moderately resistant
Daye Jicai	*Capsella bursa-pastoris*	11.11	moderately resistant
Youzhi Jicai	*C. bursa-pastoris*	1.48	resistant
Lianglihong 60	*R. sativus*	2.22	resistant
Qingfeng 76	*B. oleracea* var. *botrytis*	1.71	resistant
Aijingling 818	*B. rapa* subsp. *pekinensis*	11.11	moderately resistant

DI: disease index.

### 3.5. Sensitivity of Ab0920a to Fungicides

**Figure 4:**
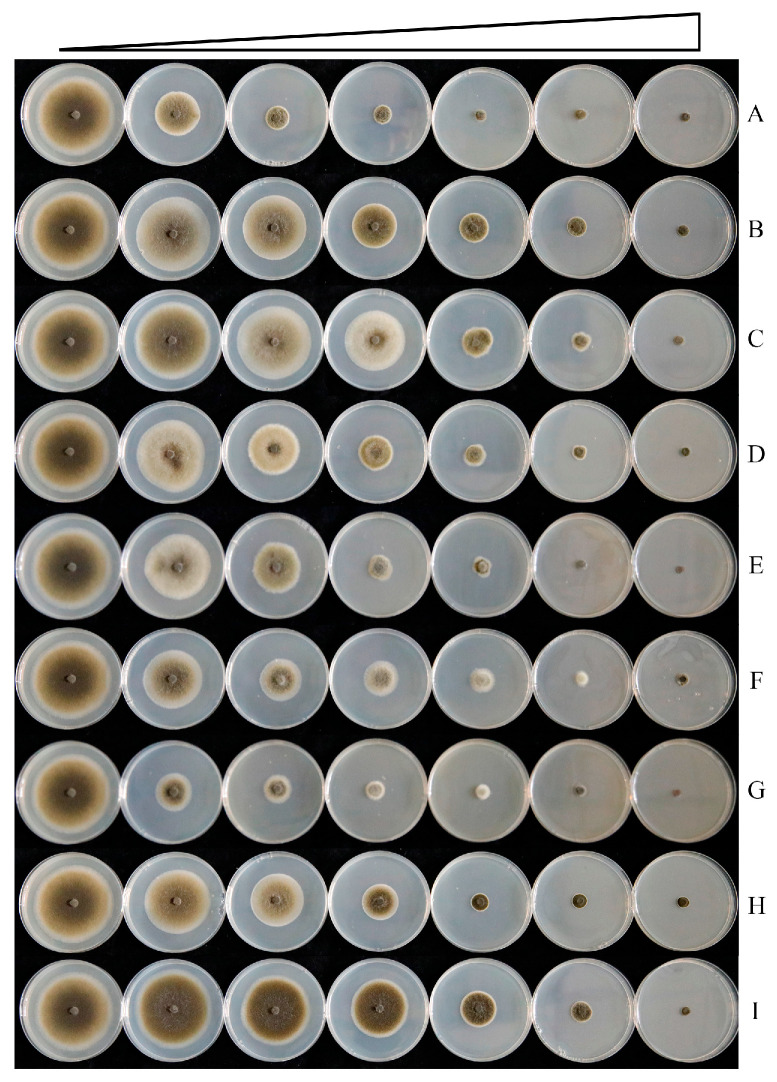
Mycelial growth of Ab0920a under different treatments/concentrations on PDA medium. The fungal growth rate of Ab0920a on PDA media plates for 7 days in the absence (check plate, column 1) or presence of different concentrations of fungicides (columns 2–7). Rows ‘A’ to ‘I’ correspond to the following treatments: prochloraz, difenoconazole, epoxiconazole, hexaconazole, tebuconazole, fluopyram, fluxapyroxad fluazinam, and iprodione.

The sensitivity of *A. brassicicola* isolate Ab0920a to nine fungicides was evaluated based on EC_50_ values. Among the tested fungicides, fluxapyroxad exhibited the strongest inhibitory activity, with an EC_50_ value of 0.0695 µg/mL. Prochloraz and difenoconazole also showed potent activity, with EC_50_ values of 0.0711 µg/mL and 0.0863 µg/mL, respectively. Tebuconazole, hexaconazole, fluopyram, and epoxiconazole demonstrated moderate inhibitory effects, with EC_50_ values of 0.1186 µg/mL, 0.1684 µg/mL, 0.2073 µg/mL, and 0.2085 µg/mL, respectively. Iprodione was less effective (EC_50_ = 0.2980 µg/mL), while fluazinam showed the weakest activity among the tested compounds (EC_50_ = 0.6632 µg/mL) (Table 3, Figure 4).

### 3.6. Prediction and Functional Annotation of Secreted Proteins and Candidate Effectors

The annotated genome of *A. brassicicola*, containing 10 514-predicted protein-coding genes, was retrieved from public databases. First, we screened the full proteome for known fungal effector-related Pfam domains. This analysis identified proteins carrying pathogenesis-associated domains, including 9 CFEM (PF05730), 9 LysM (PF01476), 2 NLP (PF05630), 14 PX (PF00787), 2 CRN (PF20147), 13 Chitin_bind1 (PF00187), 6 FAD (PF00667), and 15 CCDT (PF00264) domains (Table S4). Using SignalP, 941 secreted proteins carrying canonical N-terminal signal peptides, representing 8.95% of the total proteome, were identified. These secreted proteins were further filtered to remove those with transmembrane domains (TMHMM) or mitochondrial targeting signals (TargetP) and were subjected to effector prediction using EffectorP. The pipeline yielded a final set of 237 candidate effectors. Among these candidates, 31 SCRPs, a class of proteins commonly involved in host–pathogen interactions in necrotrophic fungi, were identified (Table S5). In addition, 68 effector candidates showed considerable homology with known virulence factors in the PHI database, supporting their potential roles in pathogenicity (Table S6). Functional characterization of 237 effectors revealed conserved motifs and CAZyme annotations. Conserved motif scanning detected several effector-associated sequence patterns, including seven proteins with the RXLR motif, 77 with the [Y/F/W]xC motif, seven with the G[I/F/Y][A/L/S/T]R motif, 26 with the [L/I]xAR motif, two with the YxSL[R/K] motif, and three with the LFLAK motif (Table S7). Carbohydrate-active enzyme annotation showed that 69 candidates belonged to diverse families, including 14 AA, seven CBM, 12 CE, 18 GH, one GT, and 14 PL, indicating their potential involvement in plant cell wall degradation during infection (Table S8). Collectively, these results provide a comprehensive inventory of the secretome and candidate effectors of *A. brassicicola*, forming a foundation for future functional characterization of key virulence factors.

### 3.7. Secretion Validation via the pSUC2 Yeast System of Isolate Ab0920a

**Figure 5:**
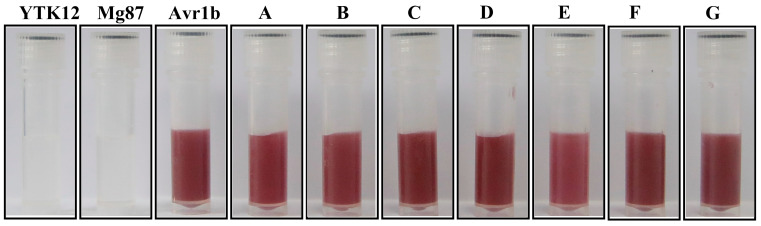
Secretion assay of candidate effectors from *A. brassicicola.* Yeast strain YTK12 carrying the empty vector and Mg87 (non-secreted negative control) showed no color change. The strain expressing Avr1b was used as the positive control, exhibiting a distinct red color. Tubes A–G represent strains expressing candidate effectors of *A. brassicicola*, all of which displayed a positive red reaction, indicating that these proteins are secreted.

Secretory activities of the selected candidate effectors were validated using the pSUC2 yeast invertase secretion system. Consistent with the expected results, the positive control (pSUC2-Avr1b) exhibited robust growth on the YPRAA medium (sucrose as the sole carbon source) and yielded distinct red colonies in the TTC staining assay, confirming efficient signal peptide-mediated invertase secretion. In contrast, the negative control (pSUC2-Mg87) failed to grow on the YPRAA and remained unstained, verifying the specificity of the assay. Notably, all transformants carrying the signal peptide-encoding regions of the selected candidate effectors displayed growth phenotypes on the YPRAA medium identical to those of the positive control, and all colonies turned bright red following TTC staining (Figure 5). These results demonstrate that the N-terminal signal peptides of all tested candidate effectors were functionally capable of mediating protein secretion in the yeast system, thus confirming all selected candidates as secreted proteins.

### 3.8. Pvx-Based Functional Validation of Effector Regulation on Bax-Induced Cell Death of Isolate Ab0920a

**Figure 6:**
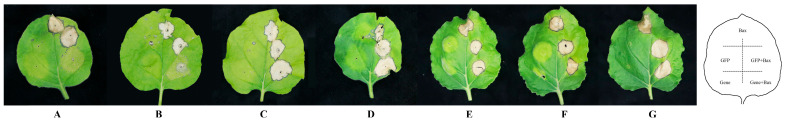
PVX-Bax cell death assay in *N. benthamiana* leaves. *Agrobacterium tumefaciens* strains carrying the PVX binary vector expressing the indicated genes were infiltrated into *N. benthamiana* leaves. The photograph was taken at 5–7 dpi. (**A**–**G**) Independent candidate effectors.

The ability of the seven candidate effectors to modulate Bax-induced cell death was evaluated using Agrobacterium-mediated transient expression in *N. benthamiana.* As expected, the positive control (empty pGR107 + pGR107-Bax) induced clear hypersensitive response (HR)-like cell death in the infiltrated leaves from 3 dpi, with severe necrosis observed at 7 dpi. In contrast, the negative control (empty pGR107 alone) did not trigger any cell death throughout the experimental period, confirming the specificity of the assay (Figure 6). Among the seven candidate effectors tested, two (designated jgi|Albra1|97181|ABRCTG0.237_pred_mRNA_4 and jgi|Albra1|100878|ABRCTG3.149_pred_mRNA_4) significantly suppressed Bax-mediated cell death when co-expressed with Bax (Table S9). Infiltrated leaves co-expressing these two effectors and Bax showed minimal or no necrosis even at 7 dpi, with phenotypes similar to those of the negative control. The remaining five candidate effectors failed to inhibit Bax-induced cell death; their co-expression with Bax resulted in HR-like necrosis comparable to that of the positive control. These results indicate that the two identified effectors possess the functional capacity to suppress Bax-mediated programmed cell death in *N. benthamiana*, suggesting their potential role in modulating host immune responses during pathogen infection.

## 4. Discussion

In this study, the pathogenic characteristics, molecular identification, host range, and effector functions of *Alternaria brassicicola* isolate Ab0920a, a causal agent of broccoli leaf black spot disease, were systematically investigated. These results provide critical insights into the interactions between this necrotrophic pathogen and cruciferous crops. First, field investigations revealed an 85% disease incidence rate, with typical circular to fusiform lesions on leaves and stems, followed by necrosis and spore production. These symptoms were consistent with previous descriptions of *A. brassicicola* infections in *Brassicaceae* crops [1], confirming the strong adaptability and destructive potential of this pathogen under natural field conditions. This high disease incidence highlights the urgency of developing effective control strategies and screening for resistant germplasm resources for agricultural production. Morphological and molecular identification collectively validated the isolate Ab0920a as *A. brassicicola*. The PDA colony characteristics (white cottony mycelia turning brown, velvety sporulation) and conidial features (muriform, obclavate to ellipsoid, and 2–8 transverse septa) aligned with the taxonomic criteria of the genus *Alternaria* [46]. Multilocus phylogenetic analysis using *Alt a1*, *ATPase*, *CAL*, and *GPD* further confirmed its close evolutionary relationship with the reference strain *A. brassicicola* EEB 2232 (bootstrap value = 100). This multilocus approach enhances identification accuracy compared with single-gene sequencing, as it reduces the impact of intra-species sequence variation and horizontal gene transfer [26]. The deposited GenBank sequences (PZ399336–PZ399339) also provide valuable molecular resources for future pathogen detection and studies on population genetics.

Pathogenicity assays on 27 cruciferous varieties demonstrated a broad host range for Ab0920a, with significant variations in resistance levels among varieties and crop species. For example, Chinese cabbage exhibited diverse reactions from highly resistant (HR) to highly susceptible (HS), whereas cauliflower and cabbage were predominantly resistant (R) to susceptible (S). This variation reflects the genetic diversity of host resistance mechanisms and co-evolutionary dynamics between *A. brassicicola* and its hosts. Our pathogenicity assay revealed marked differential resistance among 27 cruciferous varieties, consistent with previous observations that *Brassica* germplasm exhibits quantitative resistance to *A. brassicicola* [1]. Notably, the resistant varieties, such as ‘Lvxiu 100’ and ‘Youzhi Jicai’, may possess elevated epicuticular wax content or enhanced JA/ET signaling, as recently demonstrated in broccoli by Gangurde et al. [9], where wax composition and hormone crosstalk were shown to contribute significantly to resistance. These resistant materials provide valuable genetic resources for breeding, but their field performance and underlying QTLs require further investigation.

Our sensitivity data showed that fluxapyroxad (SDHI) and prochloraz/difenoconazole (DMIs) were the most active in vitro, with EC_50_ values below 0.1 µg/mL. These values are comparable to baseline sensitivities reported for *A. brassicicola* from broccoli seeds in Georgia, USA [8]. However, it has recently been demonstrated that field isolates from infested seeds can develop cross-resistance between different SDHI fungicides, underscoring the need for routine monitoring [11]. The relatively weak activity of fluazinam (EC_50_ = 0.6632 µg/mL) aligns with previous reports in Alternaria spp. and may reflect intrinsic low affinity or pre-existing tolerance [26]. Tebuconazole, hexaconazole, fluopyram, and epoxiconazole exhibited moderate effects (EC_50_ = 0.1186–0.2085 µg/mL), while iprodione was less effective. Overall, our results provide a local baseline for Shanghai isolates, but rotational use of SDHI and DMI is advised to delay resistance selection, particularly given the risk of cross-resistance documented in seed-borne populations [11].

The prediction and functional annotation of secreted proteins and candidate effectors shed light on the virulence mechanisms of Ab0920a. Among the 237 candidate effectors, 68 showed homology to known virulence factors in PHI-base, and 31 were classified as small cysteine-rich secreted proteins (SCRPs), which are well documented in necrotrophic fungi for suppressing host immunity and promoting colonization [47]. Notably, Cramer and Lawrence (2004) identified 43 *A. brassicicola* genes specifically upregulated during *Arabidopsis* infection, including putative cell wall-degrading enzymes and transporters [6]. Our genome-wide prediction overlaps with that list (e.g., GH family members), reinforcing the relevance of these candidates to actual pathogenesis. Moreover, the presence of diverse CAZyme families (AA, GH, PL, and CE) alongside phytotoxin-biosynthesis genes (Pedras et al., 2009) suggests a dual strategy—toxin-induced necrosis and enzymatic maceration—that is typical of necrotrophs [7,21]. This combined arsenal likely facilitates rapid tissue colonization and nutrient acquisition, hallmarks of *A. brassicicola* infection.

In this study, multiple conserved motifs associated with pathogenicity, including RXLR, [Y/F/W]xC, G[I/F/Y][A/L/S/T]R, [L/I]xAR, YxSL[R/K], and LFLAK, were identified in the predicted effector repertoire of isolate Ab0920a. These motifs have been widely documented across diverse plant pathogenic microorganisms and are known to play critical roles in effector secretion, host translocation, and the suppression of plant immunity. For instance, the RXLR motif serves as a canonical host cell entry signal in oomycetes and certain fungi, facilitating the translocation of effectors into the plant cytoplasm to interfere with defense signaling pathways [25,43,48]. Similarly, the [Y/F/W]xC motif was initially characterized in haustorium-specific effectors of powdery mildew and rust fungi, suggesting a conserved function in obligate biotrophic pathogens [49,50]. Although the G[I/F/Y][A/L/S/T]R and [L/I]xAR motifs are relatively conserved among fungal effector families, they remain functionally uncharacterized in most systems and warrant further investigation. The YxSL[R/K] motif has been shown to be significantly enriched in candidate effectors of *Phytophthora* and *Pythium* species, implying a potential cross-species role in pathogenesis [51]. The LFLAK motif is commonly recognized as the N-terminal signature of CRN effectors, which are involved in regulating plant cell death, further supporting the possible functional relevance of CRN-like effectors in our isolate [52]. Although the presence of these motifs alone is insufficient to confer pathogenicity, their collective enrichment provides a multifeature signature for prioritizing candidate virulence factors. Notably, substantial sequence variations may exist for some motifs across different strains, underscoring the necessity of integrating transcriptional profiling and functional validation to elucidate their precise roles in pathogenesis.

Functional validation of effector secretion using the pSUC2 yeast system demonstrated that the N-terminal signal peptides of all the tested candidate effectors were competent for protein translocation into the endoplasmic reticulum, thereby ensuring their extracellular delivery during infection. This secretory capacity is a prerequisite for effectors to reach the apoplast or gain entry into host cells, where they may interact with plant immune components to facilitate infection [41]. The successful secretion of all tested candidates suggests that the in silico signal peptide predictions are reliable and that these effectors are likely deployed at the host–pathogen interface during pathogenesis.

In a PVX-based functional assay, two effectors were identified as suppressors of Bax-induced programmed cell death (PCD) in *N. benthamiana*. Bax, a pro-apoptotic protein in mice, triggers cell death in plants that phenocopies the hypersensitive response, a hallmark of plant innate immunity [53]. The ability of these two effectors to inhibit Bax-induced PCD implies that they may interfere with conserved host immune signaling pathways downstream of or converging with HR execution. This suppression of host cell death is particularly relevant for necrotrophic pathogens, which typically benefit from host tissue death to acquire nutrients [54]. However, the remaining five candidate effectors that failed to suppress Bax-induced cell death may perform other virulence functions, such as facilitating tissue colonization, detoxifying host antimicrobial compounds, or modulating other branches of plant immunity that are not coupled to HR. Alternatively, they may require specific host factors that are absent in the heterologous *N. benthamiana* system or may act redundantly with other effectors.

Collectively, validation of pSUC2-based secretion confirmed that extracellular delivery is a common feature of these candidates, while the differential capacity to suppress Bax-triggered cell death highlights the functional diversification among effectors. These two Bax-suppressing effectors represent promising starting points for elucidating the molecular mechanisms by which *A. brassicicola* subverts the host immune response. Future studies should focus on identifying host protein targets and elucidating whether they act directly on HR signaling components or indirectly by modulating cellular processes that influence cell death outcomes.

Despite these advances, this study has several limitations that warrant further investigation. First, the pathogenicity assay was conducted under controlled conditions, and the field resistance of the identified HR varieties requires validation under natural environments. Second, the functional characterization of the candidate effectors was limited to seven proteins; high-throughput screening of the remaining 237 effectors is required to identify additional virulence factors. Third, the genetic basis of host resistance remains unclear. Comparative transcriptomic or genomic studies between resistant and susceptible varieties may facilitate the identification of key defense-related genes.

In summary, this study identified *A. brassicicola* isolate Ab0920a as the causal agent of broccoli leaf black spot disease, characterized its morphological and molecular features, and demonstrated its broad host range across 27 cruciferous varieties. The prediction and functional validation of the secreted proteins and candidate effectors revealed diverse virulence-associated factors, including SCRPs, CAZymes, and two cell death-suppressing effectors. These findings not only enhance our understanding of the pathogenic mechanisms of *A. brassicicola* but also provide valuable resources for the development of disease management strategies, such as breeding resistant cultivars and designing effector-targeted fungicides. Future research should focus on the functional characterization of additional effectors, the genetic basis of host resistance, and field validation of resistant varieties to effectively control *Alternaria* leaf black spot disease in cruciferous crops.

## Figures and Tables

**Table 3:** Sensitivity of *A. brassicicola* isolate Ab0920a to nine fungicides.

Fungicide	Slope ± SE	chi-Square Value (χ^2^)	EC_50_ (µg/mL)	95% Confidence Limits
Fluxapyroxad	1.2504 ± 0.0869	8.9480	0.0695	0.0513–0.0940
Prochloraz	1.3780 ± 0.0787	8.8552	0.0711	0.0556–0.0909
Difenoconazole	1.9504 ± 0.0842	8.2523	0.0863	0.0767–0.0972
Tebuconazole	1.8400 ± 0.1498	6.9697	0.1186	0.0924–0.1522
Hexaconazole	1.9550 ± 0.2121	6.8707	0.1684	0.1275–0.2224
Fluopyram	1.6702 ± 0.1774	8.5086	0.2073	0.15443–0.2785
Epoxiconazole	2.3129 ± 0.1367	5.9826	0.2085	0.1781–0.2440
Iprodione	1.8579 ± 0.0893	6.8095	0.298	0.2706–0.3282
Fluazinam	1.0274 ± 0.1002	8.4912	0.6632	0.4472–0.9834

Slope ± SE: slope of the probit regression line (change in probit response per log_10_ concentration) ± its standard error; χ^2^: chi-square statistic for goodness-of-fit of the probit model; EC_50_ (µg/mL): median effective concentration, i.e., the fungicide concentration that inhibits 50% of the pathogen; 95% confidence limits: lower and upper bounds of the 95% confidence interval for EC_50_.

## Data Availability

The data is not publicly available. Anonymized data may be provided upon request from the principal authors.

## Supplementary Materials

The following supporting information can be downloaded at: https://www.mdpi.com/article/10.3390/life16071099/s1, Figure S1: Workflow and functional annotation of candidate effectors predicted from the *A. brassicicola* genome. Table S1: Primers for *Alt a1*, *ATPase*, *CAL*, and *GPD* genes. Table S2: Candidate effectors for verification of secretory function and Bax inhibition. Table S3: Primers used for verifying Bax inhibitory activity of candidate effectors. Table S4: Functional domain-based classification of *A. brassicicola*. Table S5: Conserved domain annotation of SCRPs from *A. brassicicola* using the CDD database. Table S6: PHI-base annotation of putative virulence factors in *A. brassicicola*. Table S7: Identification of conserved effector motifs in predicted secreted proteins of *A. brassicicola*. Table S8: Consensus prediction of carbohydrate-active enzymes (CAZymes) in *A. brassicicola*. Table S9: Predicted effector candidates and their key functional traits.

## Abbreviations

The following abbreviations are used in this manuscript:

*Alt a1*	*Alternaria alternata* major allergen 1
*GPD*	glyceraldehyde-3-phosphate dehydrogenase
*ATPase*	adenosine triphosphatase
*CAL*	calmodulin
PDA	potato dextrose agar
dpi	days post-inoculation
DI	disease index
HR	highly resistant
R	resistant
MR	moderately resistant
MS	moderately susceptible
S	susceptible
HS	highly susceptible
DMSO	dimethyl sulfoxide
SE	standard error
EC_50_	median effective concentration
JGI	Joint Genome Institute
CFEM	common in fungal extracellular membrane
LysM	Lysin Motif
PX	Phox homology
CRN	Crinkler
CCDT	common central domain of tyrosinase
SCRPs	small cysteine-rich secreted proteins
PHI	the pathogen–host interactions
AA	auxiliary activities
CBM	carbohydrate-binding modules
CE	carbohydrate esterases
GH	glycoside hydrolases
GT	glycosyltransferase
PL	polysaccharide lyases
TTC	2,3,5-Triphenyltetrazolium chloride
PCD	programmed cell death
