# Supplementary material for: Genomic and Functional Characterization of an Alternaria brassicicola Isolate Causing Black Spot Disease on Broccoli Leaves
Source: Life (Basel). 2026 Jun 30;16(7):1099. doi: 10.3390/life16071099 (PMC13413155; doi:10.3390/life16071099)
Supplement: Supplementary file 1 [file life-16-01099-s001.zip › Figure S1.pdf]

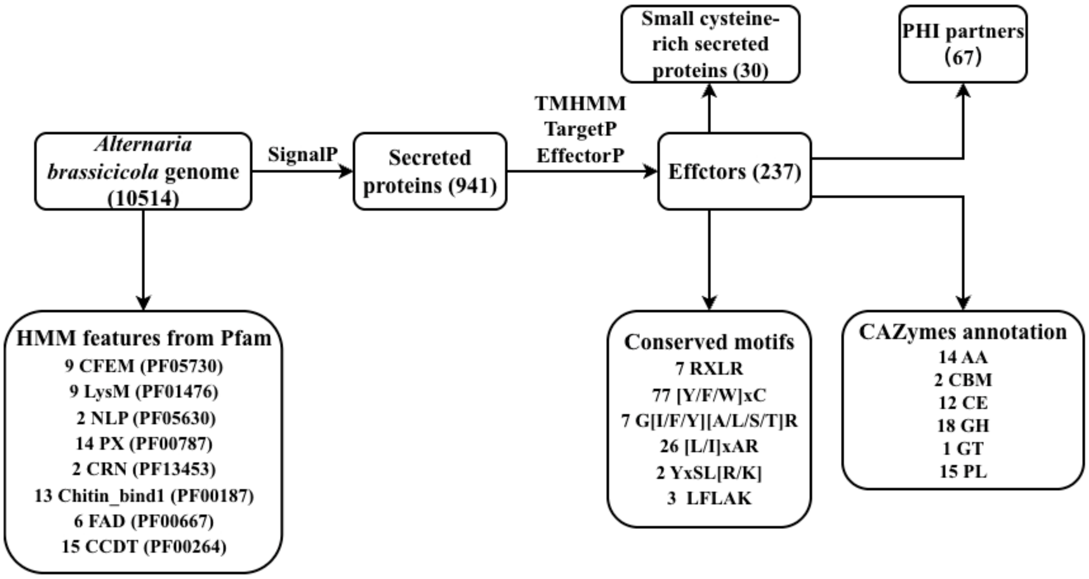

Figure S1 Workflow and functional annotation of candidate effectors predicted from the *Alternaria brassicicola* genome

A total of 10,514 protein-coding genes were analyzed. First, 941 secreted proteins were identified using SignalP. These proteins were further filtered by TMHMM, TargetP, and EffectorP to predict 237 candidate effectors. Functional annotations of the effectors included: conserved motifs (RXLR, [Y/F/W]xC, etc.), Pfam domains (CFEM, LysM, NLP, etc.), CAZymes, small cysteine-rich secreted proteins (SCRSPs), and PHI partners. The number of proteins in each category is indicated in parentheses.
