# Supplementary material for: Genomic and Functional Characterization of an Alternaria brassicicola Isolate Causing Black Spot Disease on Broccoli Leaves
Source: Life (Basel). 2026 Jun 30;16(7):1099. doi: 10.3390/life16071099 (PMC13413155; doi:10.3390/life16071099)
Supplement: Supplementary file 1 [file life-16-01099-s001.zip › Table S1.pdf]

Table S1. Primers for Alt a 1, ATPase, CAL, and GPD genes

Target	Primer Name	Primer Sequence (5'→3')	Reference
Alt a1	Alt-for	ATGCAGTTCACCACCATCGC	Hong et al., 2005
	Alt-rev	ACGAGGGTGAYGTAGGCGTC*	
ATPase	ATPDF1	ATCGTCTCCATGACCGAGTTCG	Lawrence et al., 2013
	ATPDR1	TCCGATGGAGTTCATGATAGCC	
CAL	CALDF1	AGCAAGTCTCCGAGTTCAAGG	Lawrence et al., 2013
	CALDR1	CTTCTGCATCATCAYCTGGACG*	
GPD	gpd1	CAACGGCTTCGGTCGCATTG	Berbee et al., 1999
	gpd2	GCCAAGCAGTTGGTTGTGC	

*degenerate base Y = C/T
