# Supplementary material for: Genomic and Functional Characterization of an Alternaria brassicicola Isolate Causing Black Spot Disease on Broccoli Leaves
Source: Life (Basel). 2026 Jun 30;16(7):1099. doi: 10.3390/life16071099 (PMC13413155; doi:10.3390/life16071099)
Supplement: Supplementary file 1 [file life-16-01099-s001.zip › Table S2.pdf]

Table S2. Candidate effectors for verification of secretory function and Bax inhibition

<table>
<tr><th>ID</th><th>Sequence(5’-3’)</th></tr>
<tr><td>jgi|Albra1|97181|ABRCTG0.237_pred_mRNA_</td><td>ATGCTCAACTTGGCTGTTCAGCTCCTCGCAGCCGCGAGTGTCGTCCTCGCTTCACCTGTCAACCTCCAAAGCCGTGCTGCCATCAACCATGATGCTGTCGTTGGCTTCCCCGAGACGGTCCCCTCTGGCATCGTAGGGCAGCTCATGCTCAAGTACAAGCCATTCCTCAAGGTCGACAACGGCTGTGTTCCCTTTCCTGCTGTTAACGCCGCGGGTGACACTGGGCAAGTTCTCGTCGCCGGCCTGGCCACGTCTGGTGACCCCTCAGGGATGTGCAAATCCAGCCCCGGCCAAGTCTACGCCCGCGCCAGCACCCACAAGGGCGCCTACGCCATCATGTACTCGTGGTACATGCCCAAGGACAGCCCTGGCCCTGGTCTCGGCCACACCCACGATTGGGAGAACATCGTAGTGTGGCTATCTGCCGAATCCGCCACCGCTACCATTCGTGGCGTCGCCATCTCGGCTCATGGTGATTACCAAAAAGCTACCAAGCCAAACCTCAGCGGCACTCGTCCCCTTATTGGTTACAGGTCCATTTTCCCCATCAACCACCAGCTGGTCTCTACAAGTACGAAGGGCGGAGAGCAGCCGGTGATTGCGTGGGACAGTATGCCTGCGGCGGCGAAGAAGGCAATCGAGAACACGGACTTTGGTAGCGCGATTCCTTCTTTTCGGGACAGCAACTTTGGAAGATACCTTGACGAGGCTTTCATTTAG</td></tr>
<tr><td>jgi|Albra1|100878|ABRCTG3.149_pred_mRNA_4</td><td>ATGTTCTTCACTTCAGCACTTCTCTGCCTTGGGCTGAGCGCATCGACATGTCTCGCACACTGGAACTACGACCGTATAATCGTCAATGGCGAGATTATTGGCTCACCCTACCAATATGTTCGTAAAACCAACAACTCCAACTACCCCCTTCAGAATGTCAATTCTCCACATATGCGCTGCAACTCCGGCGCCGAATCTGGCATTGCACTCAATACTCAGACTTATACTGTCGCCGCGGGTGACATGCTAGGCTTCGCAGTCAAGGATACTTTCGGCCACCCCGGACCGCAGCAAGTGTATCTTTCCAAGGCGCCTCGGTCCGCCGCTGAGTACGATGGCTCAGGCGACTGGGCAAAGATCTACAGCCTGACCTACTCATTCAACTCAAGCTATGGCGCCAGCGACGGCCTCCTTAAATGGGCGACCCACAATGCACGAACTTTCAACTTCAAGCTTTCTGCCGAAACAGCACCAGGCGAGTACCTTCTCCGCGCTGAAGGTCTAGCACTTCACGCAGCGCACAAAGCAGATAATTACCAGTTCTACGTTGCTTGCGCACAGATCAACGTCACGGGCAGTGGTGCGGGTGTTCCGGGTCCGACGATCAGGTTCCCGGGTGGTTATCAGTGGAATTCGACGGGTGTGTTGATATCAGAGTTTTGGAGCAAGATTACGAATTACACGGCCGCGGGACCAAAGTTGTGGCCGGAAGGGACGAAGGAGGCGCATGTTTTAGATGGGACTAAGAAGACTGGTGCGGACTGA</td></tr>
<tr><td>jgi|Albra1|98019|ABRCTG11.107_pred_mRNA_1</td><td>ATGTCATTCTTTTTGTCAAGCTTGCTTCTCGCCGACACCCTTCCTTCTCCTCTCCAGCCTGGCATCGTATCCAACTGCGATGAATTCTACTTTGTAAAGCCCGGCGAGTTCTGCATCGACATTGCCAACGCCAAGGGTGTCACTCTCGACGACTTCCTCAATTGGAATCCCCATGCCGGCGAGACATGCGCCAACCTACTAGCCGACACCTATGCCTGTGTTTCCATACCCGGGCACACTGCCACACCTACGAAGCTGCCGAACGGAATCGAGACGCCCCAGCCTATTCAGGTGGGCATGGTAGACAACTGCAACAAGTTCCATTTCGTCGAGGGCGGTGAGGACTGCCCAGCTATTCAGAACGAGTATGGAGTTAGCCTGGAGGATCTTGCCAAGTGGAACCCAGCGATTAGGGATGACTGCACACTCATGTGGGCGGGTACCAACCTCTGCGTGGGAGTGGTCGCATAA</td></tr>
<tr><td>jgi|Albra1|103114|ABRCTG6.14_pred_mRNA_1</td><td>ATGCAGTTCTCCAGTGCCATCTCTGCCGCCATCCTTGGCTTTGCCAGCCTTGCTTCCGCCATCACTGTCAGCTACGACACCGGCTACGACGATGGCAACCGCGCCTTGACCTCGCTCGCCTGCTCCGACGGCGCCAACGGTCTCATCACCAAGTACAACTGGCAAACTCAGGCCAACGTTGCTGGCTTCCCCAAGATCGGTGGATACATGGGTGTTGCTGGATGGAACAGCCCCCAGTGCGGAACATGCTACGGCGTCACCTACAACGGAAAGACCGTCTATGTTCTCGCTGTCGACCATGCTGCTCAAGGTTTCAACATTGCCAAGGCAGCTATGGACGAGCTTACCAACGGCCAGGCTGCAGCTCTTGGACGTATTGATGCGCAGTACGCCCAGGTTGCTACCAGCAACTGTGGATTGTAA</td></tr>
<tr><td>jgi|Albra1|105048|ABRCTG9.91_pred_mRNA_4</td><td>ATGAAGAGCGCAATCATCTTGACCGGTGTTGCTGCCTTTGCAACACAGGACCTTTGGGTCAACGGCAAAGATCAGCACGCGTCCACCAGTCCACTTGCGCAAGGCTTCCCAACAGCAACCTCGAAATGCAGTGTCCCTGCCGGTGGCACCGTAACTGTAGAGATGCATCAGCAGAATGGCGAACGCTCCTGTGCCAACGAGGCCATTGGAGGTGCCCACTACGGACACATGGGCGAGAACACCGCTGGTGGCGGCTGTTCGGACGATTACTGGGGAACAAAGGATTTGAACAAGAACTGTGGAAAGATGGACGTGAAGATCCCTACGGATCTGGCTCCAGGAGATTGTCTATTGAGAGCGGAGGCTATCGCGCTGCATGCTGCCTCTGGTGCTGGTGGCGCTCAGTTCTACGTCACCTGCTACCAAATCACAGCCACTGGTGGTGGGTCGTCTTTGCCTGCTGGCGTGTCTTTCTCTGGGGCATACAAGGCTACCGATCCTGGTATTCAAATCAAAATCTACCAAAACATTGCTTCATACGTCGCTCCTGGTCCGGCAGTCATACCAGGTGGTACTGAAGCCGTTGCTGGAAGTGTTGGATCAGCCGTCACTGCTACTGGAGGAGCTCCGGTTGCTACAGCTACCGCAACCACGATGAAGACATCTGCAGTAGCCACATCTACTGCCCCTGCGCCAACAAATGGGGGTGTTTACGATGCATGTTCGGTTGCAAAGTTTGGTCAGTGTGGAGGAAACGGGTACAGTGGATGCGAAACCTGTGCTTCGGAACTTGCCTGC</td></tr>
</table>

ID	Sequence(5'-3')
	AAAGCTGGAGGTGACTATTACTCACAGTGCGTCTGA
jgi|Albra1|101539|ABRCTG4.87_pred_mRNA_5	ATGCAGTTCACTCCCTTGACTCTTGCCGCGCTCATTGCGTCCCTGGCTTCCGGCCAGACTCTGAACATTCCTGCTAGAGTGGGCAACGTTCAGCTTGCTAC<br>CAACACTCGCATCACTGCAAACAGGGATTTCGGCATGGCGGAGTTTGACAGTGGCATTACCTGCAACCAGGAGGCCAATGGCGACCCAGTCTTCATTCTC<br>GAAGACGGTGTCACTATTTCCAACTTGATCATTGGACCTAACCAGATCGATGATGCTGTTACCGCTCTTGGACCTGGTGATGTCCTCATCGAGGGCGGCG<br>GTGCTACTGGCGCCAACGACAAGGTCATCCAGCACAACGGACGTGGCCGCGTTACTATCCGCAACTACACCGTAACTAACTCTGGAAAGCTCTACCGCA<br>GCTGCGGAAACTGCAGCAACAACCAGGCCAACAGCCCTCGCAGTGTCGTGGTTGAGAATGTTCGCGCCAGCGGCATGACCTCTGACCTCGTTGCTATGA<br>ACCCCAACTTCGGCGACACTGCAACCATCACCCGCTCCTGCGGTAGCAACAGCCGCGTTGTCTGCCAGCCTTACACAGGTGTCGAGCGTGGCCAGGGCG<br>ATGGCCCTAAGATGGACAACAAGGATGGATGCCTTGGTGCTCAAGGACTCCTCAACAGGCTGCCCGCTTGCTAA
jgi|Albra1|102112|ABRCTG4.260_pred_mRNA_6	ATGAAGTTCAGCGCACTTCTCATCGCCAGCACGGCATCCCTCGCCCTCGCCGTACCCACCGCCACCATCCAGAAGCGTGCCGACTACTGCGGTCAATGGG<br>ACAGCACCGTGACTGGTGACTACACCGTCTACAATAACCTTTGGGGCCAAGGCAATGCCGATTCCGGCTCCCAGTGCACCGGCGTTGACGGCCTGAGTG<br>GCAGGGCCCTCAAGTGGCACACGTCGTGGTCCTGGACCGGCGGTCCCGGACACAAAGCCCTCTCCTCGGTCAAATCCCTTCCCTCAGTCTGGAAGTGGAC<br>CTACGCATGCGACAGCCTCATCGCAAACGTCGCCTACGACCTCTTCACCTCTTCAAAGGTCGACGGCGCCCCGGAGTACGAGATCATGATCTGGGTTGGT<br>GCTCTCGGCGGCGCTGGCCCCATTTCCTCTACGGGAAGCCCCATTGCTACCGTCAACCTCGCCGGCAACAGCTGGAAGTTGTACAACGGACAGCATAGCC<br>AGATGAACGTCTTCAGCTTCGTTGCGGAGAGGCAGGTCAACAGCTTCAATGGCGATTTGATGACGTTTGTTAACGAGTTGACGAGCAAGCATGGAATGC<br>CTACCAGCCAGATCTTGACTAGCGTTGGCGCTGGAACTGAGCCCTTTTCTGGTAGCAACGCGAAGTTTACTGTTACCGAGTACAGCCTGAGCCAGAGCTA<br>G

Nucleotide sequences of signal peptides from candidate effector factors are highlighted in green. EcoRI and XhoI restriction sites are added to the 5' and 3' ends respectively, followed by ligation into the pSUC2 vector.
