# Supplementary material for: Genomic and Functional Characterization of an Alternaria brassicicola Isolate Causing Black Spot Disease on Broccoli Leaves
Source: Life (Basel). 2026 Jun 30;16(7):1099. doi: 10.3390/life16071099 (PMC13413155; doi:10.3390/life16071099)
Supplement: Supplementary file 1 [file life-16-01099-s001.zip › Table S3.pdf]

Table S3. Primers used for verifying Bax inhibitory activity of candidate effectors

ID	Sequence(5'-3')
97181-bax-F	atcgatATGCTCAACTTGGCTGTTCAGC
97181-bax-R	gtcgacCTAAATGAAAGCCTCGTCAAGGTAT
100878-bax-F	atcgatATGTTCTTCACTTCAGCACTTC
100878-bax-R	gtcgacTCAGTCCGCACCAGTCTTCTT
98019-bax-F	atcgatTGTCATTCTTTTTGTCAAGCTTG
98019-bax-R	gtcgacTTATGCGACCACTCCCACGCA
103114-bax-F	atcgatATGCAGTTCTCCAGTGCCATC
103114-bax-R	gtcgacTTACAATCCACAGTTGCTGGTA
105048-bax-F	atcgatATGAAGAGCGCAATCATCTTGA
105048-bax-R	gtcgacTCAGACGCACTGTGAGTAATAG
101539-bax-F	atcgatATGCAGTTCACTCCCTTGACT
101539-bax-R	gtcgacTTAGCAAGCGGGCAGCCTG
102112-bax-F	atcgatATGAAGTTCAGCGCACTTCTCAT
102112-bax-R	gtcgacCTAGCTCTGGCTCAGGCTGTA
