# Supplementary material for: Genomic and Functional Characterization of an Alternaria brassicicola Isolate Causing Black Spot Disease on Broccoli Leaves
Source: Life (Basel). 2026 Jun 30;16(7):1099. doi: 10.3390/life16071099 (PMC13413155; doi:10.3390/life16071099)
Supplement: Supplementary file 1 [file life-16-01099-s001.zip › Table S9.pdf]

Table S9. Predicted Effector Candidates and Their Key Functional Traits

ID	Length	Description	Notes	Signal Peptide Secretion Function	Inhibition of Bax-induced Necrosis
jgi|Albra1|97181|ABRCTG0.237_pred_mRNA_4	239	necrosis inducing protein	PHI(reduced_virulence), NLP	Yes	Yes
jgi|Albra1|100878|ABRCTG3.149_pred_mRNA_4	254	glycoside hydrolase	PHI(effector), AA9, GH	Yes	Yes
jgi|Albra1|98019|ABRCTG11.92_pred_mRNA_7	156	LysM domain protein	LysM	Yes	NO
jgi|Albra1|103114|ABRCTG6.14_pred_mRNA_1	140	Cerato-platanin	PHI(reduced_virulence), SCRP	Yes	NO
jgi|Albra1|105048|ABRCTG9.91_pred_mRNA_4	277	endoglucanase II	AA9	Yes	NO
jgi|Albra1|101539|ABRCTG4.87_pred_mRNA_5	223	pecate lyase	PHI(reduced_virulence),PL	Yes	NO
jgi|Albra1|102112|ABRCTG4.260_pred_mRNA_6	232	glycoside hydrolase family 12 protein	PHI(effector),GH12	Yes	NO
